# Targeting signal transduction pathways of cancer stem cells for therapeutic opportunities of metastasis

**DOI:** 10.18632/oncotarget.10942

**Published:** 2016-07-29

**Authors:** Waqas Iqbal, Saleh Alkarim, Ahmed AlHejin, Hasan Mukhtar, Kulvinder S Saini

**Affiliations:** ^1^ Embryonic and Cancer Stem Cell Research Group, Department of Biological Sciences, King Abdulaziz University, Jeddah, Saudi Arabia; ^2^ Department of Dermatology, University of Wisconsin Medical Sciences Center, Madison, WI, USA; ^3^ School of Biotechnology, Eternal University, Baru Sahib, Himachal Pradesh, India

**Keywords:** stem cells, cancer, metastasis, therapeutic, signaling

## Abstract

Tumor comprises of heterogeneous population of cells where not all the disseminated cancer cells have the prerogative and “in-build genetic cues” to form secondary tumors. Cells with stem like properties complemented by key signaling molecules clearly have shown to exhibit selective growth advantage to form tumors at distant metastatic sites. Thus, defining the role of cancer stem cells (CSC) in tumorigenesis and metastasis is emerging as a major thrust area for therapeutic intervention. Precise relationship and regulatory mechanisms operating in various signal transduction pathways during cancer dissemination, extravasation and angiogenesis still remain largely enigmatic. How the crosstalk amongst circulating tumor cells (CTC), epithelial mesenchymal transition (EMT) process and CSC is coordinated for initiating the metastasis at secondary tissues, and during cancer relapse could be of great therapeutic interest. The signal transduction mechanisms facilitating the dissemination, infiltration of CSC into blood stream, extravasations, progression of metastasis phenotype and angiogenesis, at distant organs, are the key pathologically important vulnerabilities being elucidated. Therefore, current new drug discovery focus has shifted towards finding “key driver genes” operating in parallel signaling pathways, during quiescence, survival and maintenance of stemness in CSC. Understanding these mechanisms could open new horizons for tackling the issue of cancer recurrence and metastasis-the cause of ~90% cancer associated mortality. To design futuristic & targeted therapies, we propose a multi-pronged strategy involving small molecules, RNA interference, vaccines, antibodies and other biotechnological modalities against CSC and the metastatic signal transduction cascade.

## INTRODUCTION

Metastasis is the result of dissemination of primary cancer tissue cells that go on to initiate and colonize at distant secondary tissue sites. Cancer cells purportedly disseminate from tumors in hoards and establish new tumors in distant organs [1, 2]. Metastasis remains the leading cause of cancer related deaths in over 90% of patients. The ability to metastasize and overcome the adversaries coming their way have recently been attributed to cancer cells with tumor initiating or cancer cells with stem cell like properties better known as cancer stem cells (CSC). Understanding CSC in the light of key signaling pathways that ostensibly are the driving forces behind metastasis is of utmost clinical importance for drug discovery and development.

Tumor comprises of heterogeneous population of cells. This intratumoral heterogeneity leads to an organized hierarchy corresponding to the spatial structure of a normal tissue, self-renewing cancer stem cells, progenitor cells and tumor cells. The identification of CSC within a subpopulation with enhanced tumor initiating and growth promoting cells has led to major improvements in our understanding about how individual tumor's cellular architectural components might be vulnerable for targeted therapies. Self-renewal, invasiveness and tumor progression that were once attributed to tumor cells in general have been tracked down to a fraction of cancer cells within the tumor bulk. Hence, the role of CSC in metastasis is understandably significant and therapeutically remains a challenge. Tumor initiation by disseminated cancer cells depends on their ability to self-renew and initiate metastatic tumors, the very same properties that are attributable to CSC are intrinsic to cancer metastasis. Presence of CSC, initially identified in hematopoietic cancers [3, 4] has now been recognized in many other solid tumors, like, brain [5] [6], colon [7] [8] [9] [10] [11], breast [12–14], skin [15, 16], prostate [17], and pancreas [18] [19]. These studies have led to the recognition of CSC hypothesis *en masse*. The documentation of clinically significant metastatic genes and properties has enhanced the biological understanding of metastasis and its distinctive stages [20, 21]. High-throughput sequencing studies have been able to provide additional evidence regarding the contribution of epigenetic changes that exacerbates cell renewal and survival mechanisms crucial for metastasis [22].

CSC, unlike progenitor cells are responsible for long-term tumor growth and remains recalcitrant to currently available therapeutic options. This is analogous to normal stem cells that maintain tissues homeostasis. This small population of CSC existing in tumors may well be the cause of metastases, as these cells are known to from secondary tumors in immunodeficient mouse models. The metastatic niche & EMT act in synergy with signaling transduction pathways known to regulate CSC properties and metastasis as cancer cells with stem cell surface markers are proven to promote metastasis.

Herein, we outline current knowhow and future directions of the molecular biology of metastatic tumor growth, by focusing mainly on signaling pathways that allow metastatic CSC to survive under hostile conditions, continue infiltration into bloodstream and initiate tumors at distant organs. How these pathways might provide an opportunistic window and genetically susceptible regulatory mechanisms for targeted drug discovery & development remains largely unexplored territory? It is important to mention here that our knowledge of the mechanisms that underlie metastasis are still in its infancy and the discussion in this review mainly focuses on the findings from animal models, which often are unable to mimic the processes in human patients. It is our hope that the knowledge about how CSC, CTC, EMT and other regulatory loops coalesce to form metastases will ultimately be useful in defining new therapeutic strategies.

## CSC ROLE IN METASTASIS

Metastatic CSC (mCSC) are unique in their own intricacy, intravasation of disseminated cancer cells from its primary source of origin, extravasation into different organs and covert colonization after a latent phase has clear hallmarks that normal disseminated cancer cells are not able to achieve. Not all migrating cancer cells or CSC with metastatic potential are able to initiate tumors at distant organ sites due to the fact, that most of the disseminated cells are usually gone too far down the pathway of differentiation. The mCSC either exist and extravagate or, might be derived from disseminated tumor cells, which reacquire stemness to initiate tumors in distant organs [23, 24]. Lack of accurate animal models or 3D printed human tissues limit our knowledge to unravel these intricate processes of metastasis. One unsolved puzzle is how some disseminated tumor cells manage to acquire the properties of tumor initiating cells while others do not. Perhaps accurate models to mimic the process of metastasis and lineage tracking would help in defining the delicate relationship of disseminated tumor causing cells to its primary source of origin, and how they are able to circumvent hostile forces at play at the secondary tissue's point of entry of these “never say die” cells. CSC may represent a tiny fraction of the total cellular mass of individual tumors, yet these cells may be the critical drivers of their malignant progression to form distant metastasis.

## CSC QUIESCENCE

Cancer cells with the ability to from distant tumors in other parts of body can remain dormant for years despite the removal of primary tumor by surgery or other pharmacological approaches. How disseminated CSC remain latent, and play crucial role of stromal signaling and cell-cell interactions in maintaining quiescence remains elusive. Mouse models are unable to mimic the intricate process of dormancy and reactivation that is observed in human patients, where latency can last from months to years. Based on experimental evidence on what we know so far, it is still uncertain whether metastatic niche and molecular pathways that initiate metastasis are required before, during or after metastasis, once the cells pass through dormancy. Disseminated tumor cells (DTC) found in bone marrow were shown to be in dormant stage in terms of their tumorigenic ability [25, 26] and these DTC enter G_0_ phase of the cell cycle and fail to proliferate as a potential source of tumor propagating entities. Perhaps it is a failure of these cancer cells to metastasize and form new tumors or a defense mechanism to avoid unwanted immune response when the odds are stacked up against them [27, 28]. This transition between dormancy and proliferation is intricately controlled by a network of signaling pathways, as found in few currently available experimental models. Mitogen activated kinases like p38 and ERK act in cohort, turning the tumor cells in dormant and proliferative stage respectively. This is primarily facilitated by the activation of ERK through α5-β1 integrins by the urokinase receptor (uPAR) [29, 30]. Perhaps, it is the microenvironment niche that the disseminated tumor cells interact, *via* signaling pathways that directs tumor cells on how and when to proliferative depending on the difficulties these disseminated cells might face. This was evident in breast cancer where Bone Morphogenic Protein (BMP) signals lung parenchyma to enforce dormancy, suppression of self-renewal and encouraging differentiation. Coco, a BMP antagonist suppresses BMP *via* sequestration and hence counteracts latency [31].

## CSC TARGETING

Two important caveats need to be addressed before therapies targeting CSC and mCSC could be considered. The CSC follow the same molecular blue print as normal stem cells necessitating the importance of strategies that would discriminate CSC from normal stem cells. Currently it is unclear if drugs developed to target CSC would not target normal stem cells on the pretext of increased proliferation by CSC. Understanding the genetic networks and associated cellular & environmental factors might specifically pinpoint towards the intricacies of CSC and normal stem cells, and ultimately open up a new therapeutic window for targeted therapies [32, 33]. Obviously, measuring the therapeutic potential of a drug by observing the shrinkage in tumor size might not be helpful in evaluating CSC based therapies. Keeping in mind CSC are a minority within the tumor, their elimination alone might not reduce the tumor size significantly. Hence, studies evaluating therapeutic efficacy should also emphasize on decrease in cancer recurrence or metastasis. Understanding the role and origin of mCSC in primary tumor and tumor metastasis might change the entire outlook about how cancer is perceived and whether individual gene(s) within mCSC are druggable?

## MECHANISTIC INSIGHTS IN HIERARCHICAL HETEROGENEITY

Considerable progress has been made to understand how cancer heterogeneity behaves and in unravelling of the genetic mechanisms operating during tumor development. Cancer heterogeneity in many instances seems to be due to the hierarchical organization that a tumor follows. This hierarchical tree follows the same basic principles of organ development and resembles closely to the kinetics of tumor growth. The CSC encompassing the top of this hierarchy resemble the normal stem cells in terms of phenotype and functionality with additional oncogenic mutations as tumor progresses. CSC not just self-renew their own population but also give rise to a progeny of partially or completely differentiated cells. Lineage tracking studies in mouse models provided genetic evidence that primary tumors of colon, brain and skin follow the hierarchical organization of their tissue of origin [5, 11, 15, 33]. It is still uncertain whether the metastatic tumors arising from primary tumors follow the same hierarchical organization as the long-term survival and growth of tumors rely on CSC. The evidence for this comes from clinical studies, where expression of adult stem cell markers generally correlates with poor diagnosis, prognosis and metastatic recurrence [8, 14, 34]. Cells with the potential to form nascent tumors can be isolated using stem cell markers. These cells are also found in blood of breast cancer patients. On inoculation into immunodeficient mice, these cells can cause bone, lung and liver metastases [18, 35–37].

Convincing evidence for a lineage relationship among CSC, adult stem cells and mCSC were obtained from studies on colorectal cancer. These mechanistic studies revealed that upon acquiring genetic alterations in WNT pathway, intestinal stem cells gave rise to adenomas [38]. CSC resemble stem cells which are normally found in intestinal mucosa, sustain the tumor bulk of benign tumors by giving rise to a progeny of its kind and additionally a class of transit-progeny that differentiates into the main population of these tumors [11, 33, 39]. Tumors seem to follow this hierarchical order during late-stages of colorectal cancer [8, 34, 40] and in liver cancer metastases [8, 41, 42]. Not all cancers follow this hierarchical organization, as certain melanomas do not have defined hierarchy and might follow a different route [43, 44]. However, these tumors still contain a mass of proliferative stem cells with metastatic abilities that mimic the functional and genetic properties of stem cells and these cells maintain the cancerous state and cause metastasis.

## EPITHELIAL-TO-MESENCHYMAL TRANSITION (EMT)

EMT is fundamental to embryogenesis, especially in tissue invasion and neural crest formation or gastrulation [45]. A number of transcription factors including, Snail1, Snail2, and Snail3, ZEB1 and ZEB2 (zinc-finger E-box binding factor) and Twist are involved in EMT [45] and epigenetic changes are found to play crucial role as well [46]. TGF-β is known to stimulate EMT in breast and skin cancer models [47, 48]. Tumor cells undergoing EMT lose apical-basal polarity and cell-to-cell adhesion in addition to gaining properties that facilitate migration. Pancreatic and breast cancer cells undergoing enforced expression of EMT related transcription factors exhibit stem-like properties [13, 49]. Apart from these above mentioned reasons, EMT and stem cell markers were observed to co-express in patients with tumor metastasis, [37, 50, 51] and CSC are known to occur in both epithelial and mesenchymal states. However, it is realized that EMT facilitates cell migration [52–54] but for cancer cells to proliferate and form secondary tumor at metastatic site, transitioning back to epithelial phenotype will be required. Despite EMT being an attractive model, other cell-biological programs, yet to be discovered, might co-exist in certain carcinomas, becoming the key drivers of malignancy [55].

## METASTATIC NICHE

For disseminated cancer cells, it is important to locate and proliferate in organ site(s) that would be supportive, just like stem cells in an adult tissue. Stem cells reside in specialized site termed as niches, which provide molecular and cellular signals to promote self-renewal capabilities as well as differentiation of stem cells, as and when required. Niches have been characterized in numerous tissues, like hematopoietic bone marrow, intestinal epithelium, brain and epidermis [56–59]. CSC interact with the indigenous stem cell niches in primary tumors but as they abandon the primary site, these interactions are lost. There is increasing evidence that the survival and viability of disseminated metastatic cancer cells depends on certain host mechanistic and environment cues as a niche for these cells that could be described as “sustainability niches” that invariably include specified locations, signals, various types of stromal cells and extracellular matrix proteins.

Disseminated cancer cells may end up in random locations in parenchyma but recent findings suggest a growing possibility of occupation of stem cell niches by disseminated CSC. For instance, prostate cancer cells exhibit tendency to occupy hematopoietic stem cell niche to exploit it for its own viability, growth and sustainability [60]. Other locations include areas around blood capillaries termed as perivascular niche that provides glioma stem cells with Notch, Hedgehog and PI3k activating signals [61, 62]. Melanoma cells, breast and lung cancer cells are noticeably seen around the capillaries in brain [63] where these cells flourish forming sheath that ultimately end up hijacking the nearby capillaries for its own maintenance. Disseminated cancer cells that reside around the blood capillaries in brain have been found to express L1CAM and adhesion molecules belonging to Ig family that assist its localization around the perivascular basal lamina [64]. L1CAM expression in neurons under normal circumstances is to guide axons, whereas the expression of L1CAM in numerous types of malignancies is linked to poor prognosis [65], thereby increasing the likelihood of L1CAM playing a significant role in metastasis.

## EXTRACELLULAR MATRIX (ECM)

Distant organs are prone to influence by the primary tumors, which could lead to the establishment of a pre-metastatic niche formation [66]. This has been seen in mouse models where gastrointestinal, lung and breast tumors secrete inflammatory cytokines and enzymes into the blood stream that manipulates ECM favoring metastasis [66]. Tenascin C (TNC) a hexameric glycoprotein and periostin are ECM components that play crucial role in metastatic niche in mouse cancer models. TNC is found to support stem cell functions and plays a role in metastasis as the expression of TNC in breast tumor is linked to a higher propensity towards lung metastasis [67]. Breast cancer cells with elevated expression of TNC in xenotransplantation models, are at an advantage of initiating lung metastasis after extravasation [68]. This is perhaps due to the activation of Wnt and Notch signaling pathways by TNC. TNC expression in breast cancer cells not just facilitates metastases but also increases the survivability of these cells in microenvironment niche where they invade. The eventual migration of myofibroblasts and the expression of TNC ensure the survival and growth of micro-lesions [68]. Similar to TNC, periostin too exists in stem cell niches and appears to be crucial for lung metastasizing breast cancer cells [35]. Migrating myofibroblasts in response to TGF-β express periostin that binds to stromal Wnt ligands and presents it to cancer cells. TNC and Periostin, two ECM components thus support the survivability and proliferation of metastatic initiating CSC. TNC and periostin interact with the integrins, which are present on cell surfaces and tightly bind to each other [69].

With the passage of time, tumors tend to get rigid and this stiffness is often attributed to ECM. The rigidity provided by ECM activates the expression of focal adhesion molecule (FAK) and PI3K-AKT by cancer cells that are also observed in mCSC [70]. Lysyl oxidase (LOX) an enzyme induced by hypoxia, acts as a collagen cross-linker and facilitates the stiffness of ECM, thereby, expediting the process of rigidity. In addition to these biochemical changes, LOX acts as a bait attracting myeloid cells and thereby increasing tumor size. LOX on the other hand has been implicated in forming pre-metastatic niche [71, 72]. In hypoxia, activation of HIF-1a induces the expression of procollagen lysyl oxidase (PLOD2), another enzyme that helps stabilize the collagen crosslinking as found in mouse models of undifferentiated pleomorphic sarcoma, a predominantly aggressive subtype of sarcomas [73]. PLOD2 has been implicated in facilitating the dissemination of cancer cells with poor clinical diagnosis. A recent finding revealed a concomitant action of PLOD2, prolyl hydroxylases, P4HA1 and P4HA2, in breast cancer metastases [74]. Taken together, ECM components appear to be important for survivability and metastasis of cancer cells.

**Figure 1:**
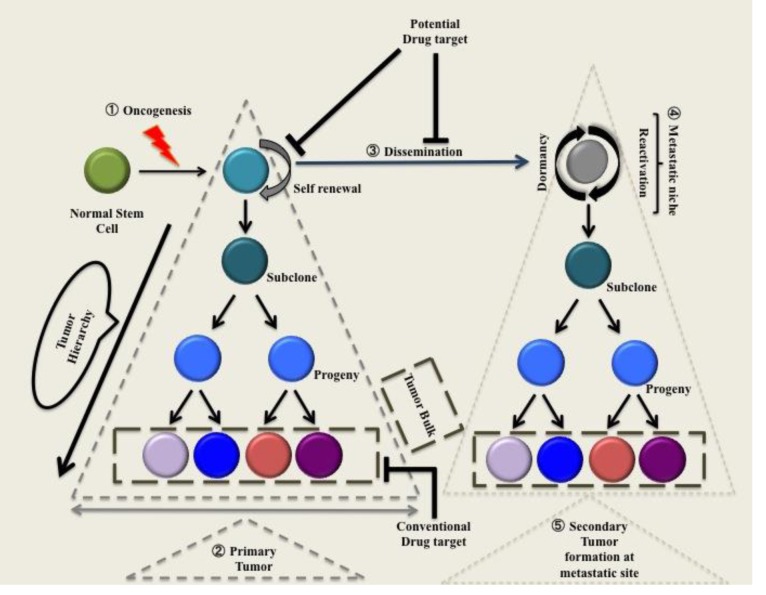
Normal stem cell after acquiring mutations in oncogenes becomes cancer stem cells 1) Normal stem cell after acquiring mutations in oncogenes becomes cancer stem cell. 2) This cell has the ability to self-renew and give rise to subclones that further give rise to a progeny that lack self-renewing capacity. These progenitor cells divide into a subset of cells that sustains the tumor bulk. 3) The disseminated cancer stem cell is dormant till it reaches metastatic niche, this increases its chances of survival upon arrival at metastatic niche. 4) Reaching metastatic niche, cancer stem cell gives rise to a secondary tumor by emulating the same vicious cycle.

Glycosaminoglycan hyaluronan is another ECM component that binds to cell surface receptor-CD44 of breast cancer cells and inhibits apoptosis during lung metastasis in mouse models [75]. The binding of ECM component glycoprotein osteopontin to CD44 receptors on cell surfaces of glioma CSC in perivascular niches enhances their metastatic aggressiveness [76]. Migration and colonization of colorectal metastatic cancer cells is also promoted by CD44 [77]. Furthermore, one of the key enzymes in hyaluronan synthesis, hyaluronan synthase-2 (HAS2) facilitates metastasis in mouse breast cancer models [78]. Elevated levels of hyaluronan are associated with clinically poor prognosis of breast cancer. Our understanding of how ECM is integral to metastases and the plasticity of mCSC would certainly increase as our knowledge broadens [79].

## SIGNALING PATHWAYS OPERATING DURING METASTASIS

Not all disseminated tumor cells that invade another tissue survive. Disseminated tumor cells should either avoid harmful stromal signals or in response, over-express anti-apoptotic & survival pathway genes and be “deaf” to death signals. This has been documented for brain metastasizing cells that up-regulate the expression of serpins to inhibit plasmin expression by astrocytes, thus preventing lethal FasL signaling [64] and circumventing apoptosis. P13K-AKT pathway contributes immensely to the survival of disseminated cancer cells. This pathway is amplified in breast cancer cells by the action of Src in the presence of CXCL12/SDF-1 and IGF1 in bone marrow [80]. The activation of Src is accomplished through estrogen receptor on luminal breast cancer cells and facilitated by CAF-rich stroma basal tumor cells [81]. VCAM1, an endothelial cell adhesion molecule, upregulates PI3K-AKT signaling in breast cancer cells, as observed during lung metastasis. VCAM1 expression in tumor cells amplifies PI3K signaling *via* Ezrin after its engagement with α-4 integrins [82]. Clinically the activity of Src in breast cancer is correlated with bone metastasis [80] whereas VCAM1 is associated with lung metastasis [67, 82]. Thus, VCAM and Src might prove to be useful biomarkers for predicting the organ specific metastatic dissemination of breast cancer cells to distant sites. Um [83] and other investigators [84] stressed the importance of pro-apoptotic members of Bcl-2 family, Bax and Bak, in suppressing cancer cell invasiveness, through the inhibition of PI3K/AKT/MMP-2 pathway [85]. Interaction of integrins with receptors, such as EGFR and Met, activates the quiescent metastasizing cancer cells in mouse models [27, 86]. Outcome of many animal studies has highlighted the role of NFκB signaling in lung, colon, and breast cancer metastasis [87, 88]. Another signaling pathway, JAK STAT3 is known to promote metastases in breast, pancreatic, prostate carcinoma and melanoma [89–91]. Most of these studies have limitations as the data obtained was based on general metastasizing cancer cells and not mCSC or CSC. Nevertheless STAT based survival of colorectal CSC metastasizing in liver has been documented. CSC invading lung or liver stimulate the production of IL-11 by stroma fibroblasts, which in turn enhances the survival through GP130/STAT3 signaling and hence promote lung and liver metastasis [92].

## MAINTAINING STEMNESS

Stem cell niches in bone marrow, intestinal mucosa and brain, promote plasticity/stemness *via* Wnt and Notch pathways [56, 58, 62]. A similar signaling is emulated by the metastatic niches in regulating mCSC. The interaction of Notch and Wnt pathways with ECM components like TNC and periostin is synonymous to how VCAM1 and Src act in abetting PI3K-AKT signaling pathway to promote survival and preserve stemness of disseminated CSC [80, 82]. In both cases, limited stromal signals at their disposal were amplified to ensure survival and proliferation of metastasizing cancer cells.

## EPIGENETIC CHANGES

CSC generally require additional set of mutations in order to successfully initiate and develop metastatic tumors. Xenotransplantation of stage IIIB/C human melanoma cells from patients with metastatic melanoma in mice has been shown to form lesions and metastasis occurred in mice with these tumor xenografts [93]. This study had an interesting observation that metastasis occurred in some of the xenotransplants from patients with non-metastatic melanoma, though the frequency was relatively low. The ability of melanoma cells to metastasize correlated with the ability of tumor cells to enter blood stream. Hence, in order to colonize & metastasize, tumor cells need to attain the ability to enter the blood and avoid signals that are catastrophic to their survival.

Recent efforts to sequence cancer genomes have further revealed extensive genetic variations within tumors from the same issue. This heterogeneity causes phenotypic variations, modulate various signaling pathways and reduce the efficacy of cancer drugs due to variable gene expression profile of these tumors. These studies have identified around 140 genes that, when modified by mutations can promote tumorigenesis [94–100].

Mutations in pathways regulated epigenetically, might also play important role in supporting and enhancing metastasis. Mutational changes in epigenetic regulators or metabolic signaling pathways (Isocitrate dehydrogenases-IDH1 and IDH2) that enhance the epigenetic signals could trigger a selection pressure, favoring proliferative and aggressive phenotypes [101, 102]. The multifaceted transcriptional outcome of epigenetic mutations in metastatic cancer cells increases the prospects of these cells to survive and proliferate even under the pressures of microenvironment they invade [22]. There is evidence showing that the aberration in the methylation of histone H3K27 enhances the transcription of VHL-HIF2a pathway, a driving force behind renal cell carcinoma [103]. These investigators further indicated that such alternations seldom affects the outlook of a primary tumor, but facilitates the expression of CXCR4 and CYTIP genes, two HIF2a genes that assist metastasis. Epigenetic suppression of Nkx2-1, GATA6, and HOPX, entities responsible for differentiation augments and enhances metastasis in non-small-cell lung carcinoma [104].

Variations in machinery regarding mRNA processing, non-coding RNAs and translation can also enhance the metastatic traits of cancer cells [21, 105–108]. Unraveling the mutations that give rise to pleiotropic alterations epigenetically would certainly enhance our knowledge of how these mutations enhance the metastatic ability of tumors. The role of miRNAs, ncRNAs, circular RNAs and other mRNA metabolism pathways involved in the initiation, progression and the development of metastatic phenotype requires integrating genetic, epigenetic and environmental cues.

## TARGETING KEY SIGNALING PATHWAYS TO COMBAT METASTASIS

### Notch pathway

Notch pathway has immense importance in terms of cell fate determination, angiogenesis, CSC and tumor immunity. Notch signaling is predominantly involved in cell-cell communication between adjacent cells through transmembrane receptors and ligands [109, 110]. This interaction of ligand on one cell with transmembrane receptor on adjoining cell initiates a two-step cleavage of the receptor; the initial proteolytic cleavage is carried out by enzymes, disintegrins and metalloproteinases (ADAM 10 or ADAM 17) also known as tumor necrosis factor-α converting enzyme (TACE). Subsequent cleavage is carried out by γ-secretase causing an intracellular fragment to detach that interacts with nuclear factors causing expression of target gene. Notch is an intricate pathway comprising of five notch ligands (Delta-like ligand 1 [DLL1], DLL3, DLL4, Jagged1 and Jagged2) and four notch receptors (Notch1, Notch2, Notch3, and Notch4), regulating a complex array of different factors. The expression of Notch receptors and ligands varies in different tumors and tumor subtypes. Moreover, post-translational modifications of Notch receptors alter their half-life and affinity towards ligands [111, 112]. On the other hand, delineated noncanonical Notch pathways are also gaining importance in cancer progression [113–117]. The diversity of Notch signaling pathway is clinically significant as targeting Notch pathway affects a heterogeneous group of cells within a tumor that includes CSC, vascular endothelial cells and immune cells. Apart from this, as discussed below under New Drug Discovery Research, understanding the role of Notch pathway in the context of tumor progression and metastasis is important in identifying new & novel targets for drug development.

**Figure 2:**
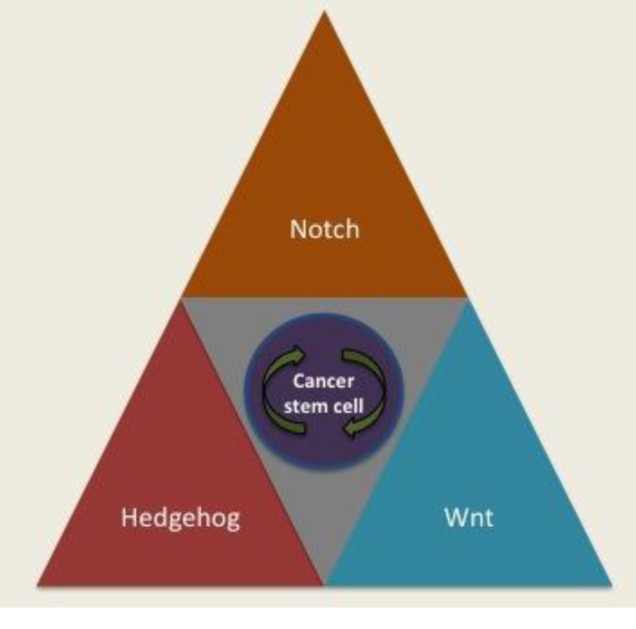
Dysregulation of Notch, Hedgehog and Wnt signaling transduction pathways in CSC is associated with the stemness Aberrations in these canonical pathways involved in self-renewal and differentiation of normal stem cells into CSC, which give them the ability to initiate tumors and promote metastasis

### Hedgehog signaling pathway

Hedgehog (HH) pathway is pivotal to tissue patterning in embryos and tissue repair as well as EMT [118]. The inhibitory effect of Patched (PTCH) transmembrane receptors on smoothened (SMO) is relieved once HH ligands (Desert Hedgehog, Sonic Hedgehog and Indian Hedgehog) bind to PTCH [119]. This activates a cascade of downstream signals initiated by SMO, leading to the activation and nuclear localization of GLI transcription factors, consequently, followed by the expression of target genes that are involved in survival, proliferation and angiogenesis [120]. This makes HH signaling a potential therapeutic target, as mutations in HH pathway lead to tumorigenesis and tumor proliferation. Such mutations could be loss-of-function mutations in PTCH1 gene that encodes Patched1 or gain-of-function mutations in SMO gene, consequently leading to ligand independent activation of downstream processes and ligand dependent downstream signals *via* paracrine or autocrine routes [120]. Activation of HH pathway caused by mutations has been observed in medulloblastoma, basal-cell carcinoma (BCC) of the skin, and less frequently in rhabdomyosarcoma [121]. Recent studies have highlighted that in 90% of BCCs and 30% of adult medulloblastomas, hyperactivation of HH pathway is associated to mutations in PTCH1 [122]. Moreover, patients with Gorlin syndrome (basal-cell nevus syndrome), an autosomal rare condition where one copy of PTCH1 gene is missing are prone to developing BCC and medulloblastoma [123]. Rhabdomyosarcomas too are thought to follow the same course though somewhat controversial due to lack of substantial supporting evidence.

The HH signaling, similar to Notch signaling pathway involves canonical and non-canonical pathways. Canonical axis involves PTCH1-SMO-GLI while non-canonical could be independent of SMO [120]. Non-canonical activation of GLI transcription factor is partially attributed to the integration of tumor-associated pathways with HH signaling [120]. Moreover, intracellular signals regulated by PI3K-AKT, KRAS-MAPK/ERK, TGF-β, IGF, inactivation of hSNF5 (a regulator of chromatin remodeling, also known as SMARCB1)andTNF-α induced mTOR/S6K1 activation have also been implicated in the activation of HH signaling pathway [124–126]. Understanding the role of tumor-associated pathways that regulate HH pathway and the interplay between them, could help develop therapies targeting HH signaling in tumor cells, CSC and their metastatic counterparts.

### Wnt signaling

There are three major pathways of Wnt-signaling cascade; canonical Wnt signaling pathway that involves β-catenin, T-cell-specific transcription factor (TCF) and lymphoid enhancer-binding factor (LEF) axis, which has been implicated in tumorigenesis and two non-canonical pathways; planar cell polarity signaling pathway that regulates the cytoskeleton and Wnt/calcium pathway, which is involved in intracellular calcium regulation. Understanding of the Wnt canonical pathway has led to increased interest in developing therapeutic strategies for its inhibition in cancer.

Inhibition of Wnt, HH and Notch signaling pathways has certainly led to the development of promising therapies that would interfere with tumor progression and metastasis (Table 1). Wnt ligands are secretory glycoproteins consisting of 350-400 amino acid residues and so far, 19 ligands have been identified in humans [127]. There are two types of post-translational modifications necessary for the secretion of Wnt ligands: binding of palmitate to a cysteine residue present at the N-terminal of the ligand [127], and serine palmitoylation in the endoplasmic reticulum mediated by Porcupine [128, 129]After secretion, ligands bind to a receptor complex that includes, Frizzled (Fz), G-protein receptor member and lipoprotein receptor-related protein 5/6 (LRP5/6). Binding of endogenous antagonist such as secreted Frizzled-related-proteins (sFRPs) and Wnt inhibitory factor-1 (WIF-1) to Wnt ligands can inhibit their interaction with receptors [130].In addition to the aforementioned antagonists, Wnt signaling pathway is regulated by inhibition of LRP co-receptors byDickkopf-related proteins (DKK) [131].Ligand binding to the receptor sends a signal *via* segment polarity protein i.e. disheveled homologue (Dvl) phosphoprotein, which is localized in cytoplasm. Once activated, Dvl inhibits β-catenin phosphorylation, mediated by Axin[132]A multiprotein “destruction complex” that includes, adenomatous polyposis coli (APC),axin andglycogen synthase kinase 3β (GSK3β), would target and degrade β-catenin in the absence of Wnt signaling. Active Wnt signaling enables accumulation and translocation ofβ-catenin to nucleus, where it activates Wnt target genes in conjunction with TCF-LEF transcription factors [133].Preclinical studies in various tumor types suggest the role of Wnt signaling pathway in maintaining CSC self-renewal [134]. It has been observed in murine models that non-melanoma cuta­neous tumor stem cells are maintained by Wnt-β-catenin cascade, perhaps in humans too [16]. Moreover, Wnt-β-catenin might play a role in EMT [135] and EMT on the other hand purportedly promotes CSC phenotype[136]. Hence, therapies targeting Wnt pathway could lead to a more potent and robust treatment options in the near future.

**Table 1:** Molecules in clinical development targeting three key signal transduction pathways of Metastasis

Notch pathway					
Condition/Tumor type	Intervention	NCT Number	Phase	Sponsor	Recruitment
Desmoid Tumors, Aggressive Fibromatosis	PF-03084014	NCT01981551	2	National Cancer Institute	Active not recruiting
Estrogen Receptor-negative Breast Cancer, Extensive Stage Small Cell Lung Cancer, HER2-negative Breast Cancer, HER2-positive Breast Cancer, Male Breast Cancer, Recurrent Breast Cancer, Recurrent Melanoma, Recurrent Non-small Cell Lung Cancer, Recurrent Small Cell Lung Cancer, Stage IV Breast Cancer, Stage IV Melanoma, Stage IV Non-small Cell Lung Cancer, Tumors Metastatic to Brain, Unspecified Adult Solid Tumor	Gamma-secretase/Notch signaling pathway inhibitor RO4929097, Radiation: Whole-brain radiation therapy (WBRT), Radiation: Stereotactic radiosurgery (SRS)	NCT01217411	1/2	National Cancer Institute	Terminated
Advanced Cancer	Notch Inhibitor	NCT01158404	1	Eli Lilly and Company	Completed
Cancer	Paclitaxel, 5-Fluorouracil (5FU), Carboplatin, Leucovorin, Irinotecan, BMS-906024	NCT01653470	1	Bristol-Myers Squibb	Active not recruiting
Hedgehog pathway					
Basal Cell Carcinoma (BCC), Basal Cell Nevoid Syndrome (BCNS)	BMS-833923 (XL139)	NCT00670189	1	Bristol-Myers Squibb, Exelixis	Completed
Prostate Cancer, Castration-resistant Prostate Cancer	Itraconazole, Orteronel	NCT02054793	1/2	Emmanuel Antonarakis, MD, Millennium Pharmaceuticals, Inc., Johns Hopkins University	Withdrawn
Prostate Cancer	Itraconazole	NCT01787331	2	University of California, San Francisco	Recruiting
Nodular Basal Cell Carcinoma	Imiquimod 5% cream with prior curettage	NCT02242929	3	Maastricht University Medical Center	Recruiting
Advanced Malignant Neoplasm, Lymphoma, Refractory Malignant Neoplasm, Solid Neoplasm	Afatinib, Akt inhibitor AZD5363, Binimetinib, Crizotinib, Dabrafenib, Dasatinib, Defactinib, FGFR Inhibitor AZD4547, Osimertinib, Palbociclib, PI3K-beta Inhibitor GSK2636771, Sunitinib Malate; Drug: Taselisib; Drug: Trametinib; Biological: Trastuzumab Emtansine; Drug: Vismodegib	NCT02465060	2	National Cancer Institute	Recruiting
Neoplasm Metastasis	LY2940680	NCT01919398	1	Eli Lilly and Company	Recruiting
Pancreatic Cancer	Gemcitabine and nab paclitaxel	NCT02358161	1/2	Academisch Medisch Centrum - Universiteit van Amsterdam (AMC-UvA), Novartis, Celgene Corporation	Recruiting
Basal Cell Carcinoma	LDE225	NCT01529450	-	Anne Chang, Novartis, Stanford University	Terminated
Wnt pathway					
Acute Myeloid Leukemia|Chronic Myeloid Leukemia	PRI-724, PRI-724, PRI-724	NCT01606579	1/2	Prism Pharma Co., Ltd.|inVentiv Health Clinical	Active, not recruiting
Carcinoma, Basal Cell	Sinecatechins 10%, Placebo	NCT02029352	2/3	Maastricht University Medical Center, Will-Pharma, Medigene AG	Completed
Advanced Solid Tumors	PRI-724	NCT01302405	1	Prism Pharma Co., Ltd., inVentiv Health Clinical	Terminated
Metastatic Breast Cancer, Colorectal Cancer, Prostate Cancer	Foxy-5	NCT02020291	1	WntResearch AB	Completed
Colon Cancer	Decitabine	NCT01882660	-	Academisch Medisch Centrum - Universiteit van Amsterdam (AMC-UvA)	Recruiting
Metastatic Breast Cancer, Metastatic Colon Cancer, Metastatic Prostate Cancer	Foxy-5	NCT02655952	1	WntResearch AB	Recruiting

Data taken from www.clinicaltrials.gov (as of 30-Apr-2016).

## STRATEGIES TO TARGET METASTASIS

Establishing a correlation between CSC and metastasis could have an immense implication on the future cancer treatment. Model experiments stress the importance of taking preventive measurements earlier than what is practiced by oncologists currently. The notion that stem cells are quiescent, seldom divide and have distinctive properties as compared to the main population of a tumor in conjunction with the ability to express higher levels of drug transporter proteins to flush out chemotherapeutic drugs, has necessitated the development of new drugs to target CSC [137]. The ability of CSC to confer resistance to radiotherapy in breast cancer cells and gliomas through increased activation of DNA damage control and repair capacity in CSC have led various investigators to hypothesize that CSC and signal transduction pathways have a major role to play in the regulation of radiation response and radio-resistance [138–140]. This necessitates the need to develop treatments that would target CSC, mCSC and related signaling cascades. The identification of key regulatory mechanisms and genetic networks that distinguish CSC from non-CSC is therefore critical for CSC-targeted therapy. Treatments targeting CSC would certainly revolutionize the way cancer therapy is presently carried out, thereby opening doors to a new perspective that could lead to more reliable targeted therapeutic modalities.

Considering that CSC are paramount to the growth of primary tumor and metastasis, it seems logical to target the self-renewal capabilities of these cells. Therapies targeting self-renewal modalities developed hitherto, cyclopamine targeting the hedgehog, exisulind, imatinib, and bromoindirubin-3'-oxime, affecting the Wnt/β-catenin signaling pathways have had varying degrees of success. On the contrary, we could argue that, inducing differentiation in CSC would technically eliminate their propensity to self-renewal. Clinically all *trans-*retinoic acid had been used as an inducer of differentiation in patients with acute premyelocytic leukemia with higher success rate. Additionally, TPA, butyric acid, vitamin D3, and DMSO have also been used for solid tumors, though using targeted remedies, for instance, PPARα activator, nerve growth factors or compounds like vesnarinone may turn out to be more effective [141]. Understanding the developmental pathways of self-renewal and differentiation of CSC would translate into more meaningful therapies compared to general inducers discussed above.

Tumor cells and CSC are inherently known to flush out drugs *via* ABC transporter genes, a group of drug transporter genes and other unknown cellular mechanisms that provide them defense against small molecule drugs. Chemotherapy and other therapeutic approaches that could inhibit the efflux of drugs out of CSC, by hindering the ABC transporter genes might lead to increased susceptibility of tumors to current or new therapies. One such attempt was made by inhibiting ABCB1 transporter with limited success, though scientists are hopeful that inhibitors of ABCG2 might turn out to be a success story in future [142].

Not all metastasizing cancer cells generate secondary tumors, and it appears that only CSC have the prerogative to undertake such a task. Hence, targeting CSC at the initial stages of metastasis could drastically reduce progression of a tumor in metastatic niche. Such has been the case in animal models where inhibiting CXCR4, a homing factor, prevented the formation of primary tumor in addition to blocking metastasis [143]. This implies the importance of identifying and characterizing metastatic-CSC's key gene regulatory networks and surrounding niches to block metastatic process. In addition, a thorough understanding of various factors involved in survival and proliferation of CSC at the secondary site, could benefit therapeutic strategy and also aid diagnosis & prognosis.

By the time a primary tumor is normally detected metastasizing cells have already been migrating to secondary site(s) or pre-metastatic niche has already been set up. Under such circumstances, blocking the reactivation of quiescent metastatic CSC could be an attractive therapeutic strategy. Such therapies are far from reality, as appropriate animal models depicting dormancy are required for further validation and proof-of-concept studies. Understanding the process of reactivation and factors associated with metastatic niche, would certainly help in developing safe and potent drugs.

## CHALLENGES AND WAY FORWARD

With the development of the Human Genome Sequence data blueprint in 2001, systematic gene expression profiling (GEP) efforts were directed towards normal and tumor tissues, to elucidate molecular signatures required to classify these tumors based on the genomics data as well as delineate various stages of carcinogenesis. In the last few years, for example, The Cancer Genome Atlas (TCGA) and other networks have reported four main subtypes of breast cancers, after detailed analyses of different genetic and epigenetic abnormalities observed in these tumors [144]. This consortium along with Vogelstein and Kinzler [145] have emphatically pointed out that in solid tumors, genetic alterations in at least three “ringleader genes” or “driver genes” appear to be sufficient to drive a “normal cell” into a clinically advanced tumor. As we know now that this process may take 20-30 years by the time a patient is clinically diagnosed with cancer. This relatively long “lag phase” has necessitated other intervention strategies, including chemoprevention, lifestyle changes, etc., among others. From new drug discovery research & development (NDDRD) point of view, targeting cancer, particularly highly metastatic subtypes, remains an insurmountable task, and throws ambiguous and multi-pronged strategic challenges for the NDDRD scientists and clinicians. Obviously, to target three different genes with one small molecule drug(s) is extremely difficult using conventional therapeutics, unless innovative approaches simultaneously targeting CSC, CTCs, metastatic cells, etc., are discovered, devised and implemented.

A combination therapy may be a way forward, where either 2 small molecules, or one small molecule and one biotechnology based drug, or more recently employing RNAi technology for targeting key driver genes [146], seems feasible in the short to medium term treatment modalities. Recently there was a report showing that in HER-2 positive breast cancer, a small molecule drug-Lapatinib when combined with Trastuzumab (Herceptin)-an antibody, a “dramatic” shrinkage of tumors occurred in just 11 days. As we know, primary tumors are “treatable” whereas CSC & metastases, particularly after colonization at secondary site(s), as and when this occurs, remain refractory to radiation and chemotherapies. Due to the lack of appropriate animal models, and additionally due to the physiological limitations in the translation of animal data into clinically late-stage metastatic tumors, NDDRD researchers are regularly encountering Achilles-heel of finding “cures” for common types of secondary cancers.

Another major challenge faced by the Pharmaceutical/Biotechnology companies is the safety and toxicity of NCEs resulting in high attrition rates during clinical drug development process. Individually or in combination-toxicogenomics, NGS and CRISPR-cas9 technologies, need to be leveraged early in drug discovery and pre-clinical development in appropriate animal models to identify and circumvent drug-induced toxicity issues. From personalized medicine point of view, how and where we can employ RNAi/CRISPR gene editing protocols for identifying, characterizing and targeting gene(s) responsible for intravasation to extravasation, dissemination and ultimate colonization of CSC & tumor cells at distant sites remains to be experimentally exploited and validated. A system biology level understanding of TCGA datasets along with better biomarkers to predict toxicity will certainly go a long way in customizing therapeutic options based on the GEP of the individual tumors. This will require collaboration and sharing of data from academic labs, industry, NGOs, regulatory bodies and other stakeholders to create systems and processes, wherein these data sets are available freely in the public domain, something on the lines of NCBI. Of particular interest will be the drugs, which fail in phase II/III and the lessons learnt, which need to be incorporated in future NDDRD programs. If successful strategies are designed and carried out after careful analyses of “repurposed drug molecules” some of these failed therapies might prove beneficial for another type of cancer, or another disease indication, based on critical evaluation of GEP datasets. Taken together, building on new mechanistic insights into metastasis, we need to make sure that future NDDRD is not done in isolation but in collaboration, where the cancer patient will be the ultimate beneficiary.
